# Atlanta Academy of Medicine

**Published:** 1879-07

**Authors:** H. F. Scott

**Affiliations:** Reporter


H. F. SCOTT, M.D., Reporter.

---

Academy called to order by the President, J. S. Todd, M.D.

President Todd reported two very interesting cases. One constituted within itself a *pathological venereal museum*, having had, successively, syphilis, gonorrhœa, stricture, orchitis and epididymitis.

Patient had a chancre one half an inch posterior to glans and on shaft of penis, and was given iodide of mercury in one half grain doses ter die.

Secondary symptoms first manifested themselves twelve months from infection. In the mean time, patient had gonorrhœa several times, of which he cured himself. Symptoms of orchitis making their appearance, Dr. T— was consulted; at this time the affected testicle measured fifteen inches in circumference. There were also present syphilitic, scaly eruptions on scrotum and other portions of body, especially marked on eyelids and ears Gave iodide of potash, thirty grains, with one-eighth grain corrosive sublimate three times a day; however, patient suffered so much, this treatment was discontinued and opium internally with iodoform ointment, substituted. Under this treatment the syphilitic eruptions vanished like magic. The testicle was skillfully bandaged, daily, by Dr. Gary. At the expiration of one week, evidences of pus being present, an incision was made and a large quantity of pus evacuated followed by immediate amelioration of symptoms; afterwards another incision was made at junction of scrotum with body, letting out another large amount of pus. Finally, a deep incision into the substance of the testicle itself was made; no sloughing of testis ensued and wounds healed nicely, leaving testis a little enlarged. There was present in this case, the most troublesome syphilitic rupia he had ever seen. Under use of iodide potash, 50 to 100 grains daily, this disappeared rapidly. Patient is now fifteen pounds heavier than ever before.

The other case was a negro woman aet. 39, with one child 15 years old. She was subject to periodical ovarian neuralgia, which was benefitted by quinine; symptoms returned in a few days and were promptly relieved with quinine; still later, there was another relapse together with pelvic peritonetis, which he treated with opiates and blisters. Learning that five years previously, she had had syphilis, though at present there were no symptoms of its presence except a slight ozœna, he administered ten grains iodide potash every three hours, and in three days there were no symptoms of peritonitis present. Improvement dated 24 hours after beginning of iodide potash. Patient was becoming rapidly worse, and thinks cure can be ascribed to nothing save iodide potash.

Dr. J. T. Johnson said, as regarded etiology of first case mentioned, no orchitis is apt to suppurate, especially is this true of syphilitic forms; thinks it originated in this case from irritation in urethra. Peritonitis in pelvis or any part of abdomen seldom disappeared so rapidly, and especially from using iodide potash. Besides, syphilitic peritonetis is a rare form and not generally recognized.

The President said his attention was directed to it on account of its rarity.

Dr. Johnson remarked that syphilitic patients were blessed with an immunity from disagreeable effects of medicines; thus the use of iodide of potassium, in such cases, is unattended by the irritation of the mucous membranes which ensues in non-syphilitic cases.

Dr. King asked if it was a fact that iodide potassium, when given to syphilitic persons, produced an eruption? This was answered in the affirmative.

Dr. Parke has known the well known action of iodide of potass on mucous membranes to be used as a test of the syphilitic or non-syphilitic nature of any particular case. Cases where such irritation followed its administration being considered "non-syphilitic," and *vice versa.*

Dr. Scott recommended early incision into testis in all cases of inflammation of that organ; said the excruciating pain in such cases was due to the swollen and inflamed parts being surrounded by the dense and non-yielding *tunica albuginea*, and that incision of the latter gave immediate relief and was entirely harmless. Thought the enlarged testicle, in case reported, was most probably due to breaking down of gummatous tumors; said these tumors could be made to undergo fatty degeneration and be rendered absorbable by the use of iodide potassium.

Dr. Johnson punctured the testis in one case of acute inflammation, with fine effect and no disagreeable consequences.

The President had been favorably impressed with Hutchinson's report on puncture of cases of acute orchitis, but his ardor had been rather dampened by reports of cases where sloughing had occurred. He had known gummatous tumors melt away early after use of iodide potash, but in case reported, large doses had no effect in reducing tumor, and besides patient was under the influence of iodide of potassium when first attacked with the orchitis.

